# The Limited Capacity of Sleep-Dependent Memory Consolidation

**DOI:** 10.3389/fpsyg.2016.01368

**Published:** 2016-09-13

**Authors:** Gordon B. Feld, Patrick P. Weis, Jan Born

**Affiliations:** ^1^Institute of Medical Psychology and Behavioral Neurobiology, University of TübingenTübingen, Germany; ^2^Centre for Integrative Neuroscience, University of TübingenTübingen, Germany

**Keywords:** sleep-dependent memory consolidation, declarative memory, working memory capacity, sleep deprivation, long-term memory

## Abstract

Sleep supports memory consolidation. However, the conceptually important influence of the amount of items encoded in a memory test on this effect has not been investigated. In two experiments, participants (*n* = 101) learned lists of word-pairs varying in length (40, 160, 320 word-pairs) in the evening before a night of sleep (sleep group) or of sleep deprivation (wake group). After 36 h (including a night allowing recovery sleep) retrieval was tested. Compared with wakefulness, post-learning sleep enhanced retention for the 160 word-pair condition (*p* < 0.01), importantly, this effect completely vanished for the 320 word-pair condition. This result indicates a limited capacity for sleep-dependent memory consolidation, which is consistent with an active system consolidation view on sleep’s role for memory, if it is complemented by processes of active forgetting and/or gist abstraction. Whereas the absolute benefit from sleep should have increased with increasing amounts of successfully encoded items, if sleep only passively protected memory from interference. Moreover, the finding that retention performance was significantly diminished for the 320 word-pair condition compared to the 160 word-pair condition in the sleep group, makes it tempting to speculate that with increasing loads of information encoded during wakefulness, sleep might favor processes of forgetting over consolidation.

## Introduction

The investigation of sleep and memory has a long standing tradition in psychology (Rasch and Born, 2013). Since Müller and Pilzecker coined the term consolidation in 1900, it has become clear that after encoding memory traces require a phase of stabilization for their long-term storage (Müller and Pilzecker, 1900; McGaugh, 2000). Ebbinghaus (1885) observed that during longer retention intervals the shape of the forgetting curve varies, he did not attribute this to sleep. Hence, it was left to Heine (1914) and Jenkins and Dallenbach (1924) to formally suggest a role of sleep for reduced forgetting. Still, until recently it was not generally accepted that sleep benefits memory (Frank and Benington, 2006). However, recent research has provided ample evidence that sleep and especially slow wave sleep (SWS) is essential for consolidation (Diekelmann and Born, 2010). Importantly, in the wake of this research it has been possible to determine boundary conditions for sleep to be effective in enhancing memory (Diekelmann et al., 2009; Feld and Diekelmann, 2015). For example, the timing (Gais et al., 2006; Schönauer et al., 2015), the amount (Lahl et al., 2008; Diekelmann et al., 2012) and the type of sleep (Yaroush et al., 1971; Plihal and Born, 1997) affect whether an enhancing effect of sleep on memory occurs. However, so far, it has not been investigated, whether sleep-dependent memory consolidation is affected by the amount of information that is encoded before sleep.

This is an important issue with essential implications for the conceptualization of the mechanisms underlying the beneficial effect of sleep on memory. Indeed, a long-standing theory in this regard is that sleep supports memory consolidation primarily by protecting new memory representations from retroactive interference, i.e., from being overwritten by more recently encoded materials, as sleep is a phase of generally reduced sensory input and equally reduced encoding of new information (Jenkins and Dallenbach, 1924; Ellenbogen et al., 2006; Mednick et al., 2011; Wixted, 2004). Independent of the amount of information that was successfully encoded, the probability that any one encoded item will be subject to interference remains stable, accordingly, the relative amount of forgetting due to interference does not change. However, the absolute amount of word-pairs that are forgotten due to interference increases proportionally with the amount of information successfully encoded. According to the passive protection theory, sleep should protect all information encoded during the preceding wake phase, and there should be no limit of capacity for the effect of sleep. In this case, only a more general limit in the amount of information that the brain can encode during wakefulness should limit the consolidation process. In other words, the benefit of sleep on memory would be expected to proportionally increase with increasing amounts of information encoded before sleep.

Alternatively, the benefiting effect of sleep has been conceptualized as an active systems consolidation process (Diekelmann and Born, 2010; Rasch and Born, 2013). This theory was spawned by the observation that neural representations of spatial memories residing in the hippocampus, are reactivated during periods of SWS following the encoding of these representations (Wilson and McNaughton, 1994; Skaggs and McNaughton, 1996; O’Neill et al., 2010). In humans, this reactivation can be prompted by presenting cues during SWS that were associated with the preceding learning experience thereby improving memory retention (Rasch et al., 2007; Rudoy et al., 2009; Schreiner and Rasch, 2014). While these studies underline the causal contribution of neural reactivations during SWS for memory consolidation they also prompted the intriguing idea that only a subset of memory representations is tagged during encoding to preferentially enter the sleep-dependent consolidation process (Born and Wilhelm, 2012). Indeed, studies in rodents and humans have provided preliminary evidence suggesting that memory consolidation during sleep is selective, implicating basically limited capacities of this process (e.g., Bendor and Wilson, 2012; McNamara et al., 2014). Thus, sleep preferentially consolidates emotional over neutral materials or information relevant to the participant’s future plans compared with irrelevant information (Payne et al., 2008; Fischer and Born, 2009; Wilhelm et al., 2011; Diekelmann et al., 2013; Javadi et al., 2015).

Interestingly, recent experiments in humans revealed that capacity limits of sleep-associated memory consolidation could be linked to those of working memory (Fenn and Hambrick, 2012). Only for participants who were allowed to sleep, the experiments showed a correlation between working memory performance as measured by the automated operation span task (OSPAN task, Unsworth et al., 2005) and the retention of word-pairs during a 10-h interval. This tempts to speculate that sleep-dependent memory consolidation is limited due to its relying on a limited working memory capacity.

In the present experiments, we investigated whether the benefitting effect of sleep on memory is subject to limitations of capacity. We contrasted the effects of nocturnal sleep on word-pair memories with the forgetting occurring during a post-encoding wake retention period of equal duration. To allow participants of the wake groups to recover sleep, all participants spent another night of undisturbed sleep before the Retrieval phase. To vary the amount of information, the participants learned word-pair lists of different length (40, 160, 320 word-pairs) and performance on an immediate recall test was used to estimate the amount of information encoded in the Learning phase. We predicted that in the case of a non-specific influence of sleep protecting memories from interference, the benefitting effect of sleep would not be limited in capacity and the absolute sleep effect would increase with increasing numbers of word-pairs encoded successfully. On the other hand, capacity limits that might originate from an active type of systems consolidation process during sleep, are expected to express themselves in a constant absolute level of sleep-dependent enhancement in memory, i.e., forgetting curves for wake and sleep groups should remain parallel. Importantly, this active systems consolidation account predicts that the sleep induced enhancement in memory should remain constant and not increase (or decrease) beyond a certain amount of information encoded before sleep.

## Materials and Methods

### Participants

We aimed for 120 participants (*n* = 60 per experiment) to be examined during 6 months sampling phase, which is at the upper end of sample sizes compared to other sleep studies (e.g., *n* = 17 per group in Schreiner and Rasch, 2014, *n* = 10 per group in Drosopoulos et al., 2007 or *n* = 24 per group in Fenn et al., 2003). Data collection was terminated after 6 months even if the sample was not complete, leading to a total of 108 healthy, non-smoking German native speakers participating in the study, who reported a regular sleep-wake cycle during the preceding 6 weeks. Inclusion criteria were a normal sleep-wake-schedule (at least 6 months before the experiment), an age range of 18-30 years, no history of neurological or psychological disorders and at least the qualification to enter higher education. Seven participants were excluded as outliers (two standard deviation from the mean recall performance in the Learning phase), resulting in 101 participants for data analysis. Out of the 101 participants, 48 participated in Experiments 1 and 53 in Experiment 2. In Experiment 1, 22 participants were assigned to the sleep group (12 female, mean age 23.45 years, age range 19-28 years) and 26 to the wake group (15 female, mean age 23.08 years, age range 19-28 years). In Experiment 2, 27 participants were assigned to the sleep group (12 female, mean age 23.19 years, age range 18-30 years) and 26 to the wake group (13 female, mean age 23.27, age range 18-30). All participants were reimbursed and provided written informed consent prior to participation. The study was approved by the local ethics committee.

### Design

Participants were randomly assigned to a group that was either allowed to sleep at home during the night after word learning took place (*sleep group*) or not allowed to sleep and had to stay in the laboratory (*wake group*). Each participant attended two sessions, scheduled at least 10 days apart. Participants of each group learned either few (*short list condition*) or many (*long list condition*) word-pairs, following a balanced cross-over design. The short list consisted of 40 word-pairs in both experiments. The long list consisted of 160 word-pairs in Experiment 1 and 320 word-pairs in Experiment 2.

### Procedure

**FIGURE 1:**
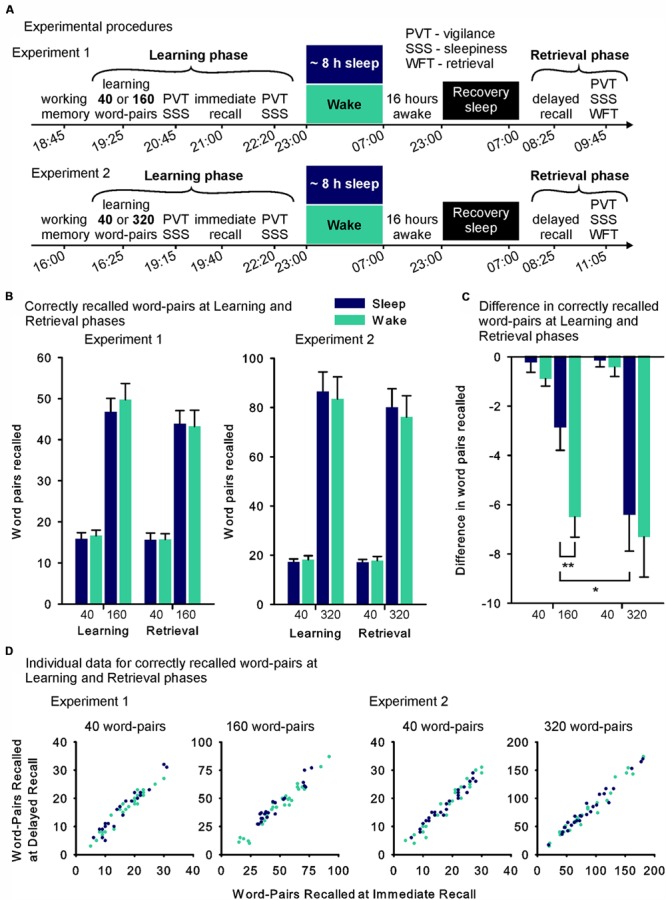
**(A)** Experimental design and time line of Experiments 1 and 2. In the Learning phase participants learned a short (40) or a long list (Experiment 1: 160, Experiment 2: 320) of word-pairs (within-subject cross-over design). During the following night, they were allowed to sleep at home (sleep group – blue fills) or stayed awake while watching animal documentaries at the lab (wake group – green fills). Roughly 36 h after the Learning phase, retrieval of the word-pairs was tested (Retrieval phase, see further explanations in the Section “Materials and Methods”). **(B)** Mean (SEM) absolute number of word-pairs correctly recalled immediately after learning (Learning phase) and at retrieval testing 36 h later (Retrieval phase) in Experiments 1 and 2 (Note that scaling of the y-axis was adapted between the experiments). **(C)** Mean differences (SEM) between performance at delayed recall during the Retrieval phase minus performance at immediate recall during the Learning phase (used as measure of retention) in Experiments 1 and 2. **(D)** Individual data for each participant’s performance during the Retrieval phase (word pairs recalled at delayed recall – y-axis) plotted against his performance during the Learning phase (word-pairs recalled at immediate recall – x-axis) for each condition in the sleep (blue) and the wake (green) groups (Note that scaling of the axes was adapted between the conditions). ^∗∗^*p* ≤ 0.01, ^∗^*p* ≤ 0.05.

Participants arrived at 18:30 h in Experiment 1 and at 15:45 h in Experiment 2 (start time was adjusted for the longer list length of the long list condition in this experiment) and sat at one of four screened work places with a computer (see **Figure 1A** for detailed times). After confirming the inclusion criteria and receiving general instructions participants performed the working memory capacity task (automated operation span task, see below). After a short break, during which participants played the computer game snood (www.snood.com), the participants learned the word-pairs (Learning phase, see also word-pair task, below). Following a short break during which participants from Experiment 2 received a small snack, participants recalled the word-pairs (immediate recall). After recall, each participant received a watch-like actimetry device (Actiwatch 2, Respironics, Murrysville, PA, USA). Participants in the sleep group were then allowed to leave the laboratory to sleep at home, whereas participants in the wake group remained under the observation of the investigator and watched a standardized set of animal documentaries. Sleep-deprived participants received two snacks during the night. They were allowed to leave the lab at 7:00 h, and were asked to abstain from sleeping until the next evening. After approximately 24 h, i.e., after the wake group had the opportunity for recovery sleep, participants returned to the lab and had to retrieve the word-pairs (Retrieval phase). All participants affirmed adherence to the sleep-wake schedule of the experiment and actigraphy data was used to confirm overall compliance (see Results).

### Word-Pair Task

The word-pair task was programmed in Presentation^®^ (version 16.3, Neurobehavioral Systems, Berkeley, CA, USA) and ran on computers running Windows 7. Before each learning or recall block, participants were instructed for the entire learning and recall procedure, including two sample trials. During learning, each pair of words was presented on the horizontal axis divided by a hyphen for 4 s (1 s inter-stimulus interval). The left word was the cue word and the right word was the target word. Word-pairs were presented in one, four and eight blocks of forty (for 40, 160, and 320 word-pair conditions, respectively) that were separated by breaks. The order of word-pairs was randomized within each block at learning and at recall and the position for each word-pair list was controlled (i.e., we balanced the position of each word-lists in the long list condition, as well as which list was used for the short list condition across participants). During the breaks participants listened to relaxing audio files and the next learning block started 20 min after the start of the previous block (a learning block lasted 3 min 20 s leaving 16 min and 40 s break until the next learning block). To keep the time lines for both list conditions identical within each experiment, in the short list condition, the participants performed a reaction time task instead of learning during all but the last block (they also listened to the same amount of relaxing audios in between) and learned the 40 word-pairs in the last block. The reaction time task consisted of arrows pointing to the left or the right and participants had to hit the right or left arrow key on a keyboard as fast as possible. Reaction times exceeding 500 ms were followed by the feedback ‘press faster’ (displayed on the screen) and faster reactions received feedback, as to whether the correct key was pressed.

The recall procedure at immediate recall (during the Learning phase) was the same as that at the Retrieval phase on the morning 2 nights later. During recall participants were shown the cue word and had a maximum of 20 s to type in the correct target word on a keyboard. Keyboard input was simultaneously displayed next to the cue word (errors could be deleted by pressing backspace). If participants were finished faster or were sure not to know the correct answer they could move to the next word-pair by pressing the return button. The recall procedure was also performed in blocks that started 20 min apart so that participants listened to varying amounts of relaxing audios between each block. A recall block lasted a maximum of 13 min and 20 s leaving at least a 6 min 40 s break until the next recall block. Participants in the short list condition again performed the reaction time task until recalling the 40 word-pairs in the last block.

Timing the beginning of each learning and recall block exactly 20 min after the beginning of the preceding block assured that learning, immediate recall after learning and retrieval of each word-pair block were separated by identical amounts of time within Experiments 1 and 2. Additionally, the break after each learning block reduced the amount of interference. For the analyses correctly recalled word-pairs were scored manually, by an investigator who was blind to the experimental conditions. Words with spelling errors or identical roots were considered correct. Absolute retention performance, i.e., the difference in correct words at recall during the Retrieval phase minus at immediate recall during the Learning phase, was used as dependent variable. (We decided not to use relative retention performance, i.e., correct words at recall during the Retrieval phase divided by those at immediate recall during the Learning phase, which is also frequently reported in memory research, as such a transformation already assumes the answer to the question at hand, i.e., that forgetting is proportional to the amount of information encoded.)

### Automated Operation Span

This computer based working memory capacity task running on E-Prime^®^ was used as provided by the authors (Unsworth et al., 2005). Participants were shown equations each followed by a letter. First the participants had to decide, if the answer provided for the equation was correct, and then remember the letter. After three to six trials they were shown twelve letters and had to click on those that had been presented before. OSPAN partial load and absolute score were used for the analyses (i.e., the sum of correctly recalled elements from all items, regardless if all elements in a trial were recalled correctly and the sum of the correctly recalled elements from only the items in which all the elements are recalled in correct serial order, respectively).

### Control Tasks

In the Retrieval phase, to exclude residual effects of sleep deprivation on general retrieval performance, participants were tested on a word generation task (Regensburger Wortflüssigkeitstest – WFT). They were asked to generate as many words as possible within a 2-min interval after being cued with either a letter (p or m as the first letter of the words to be generated) or a category (professions or hobbies). The following two control measures were assessed twice in the Learning phase (once after learning and once after the immediate recall) and once after the Retrieval phase: Mean reaction times were assessed as a measure of vigilance in a 5-min version of the psychomotor vigilance task (PVT, Dinges et al., 1997) that required pressing the space bar as fast as possible whenever a bright millisecond clock appeared on a dark computer screen and started counting upward. After the key press this clock displayed the reaction time. The mean reaction speed (i.e., 1/[reaction time in ms]) was calculated for each participant. The participants’ sleepiness was assessed with the 1-item Stanford Sleepiness Scale (SSS, Hoddes et al., 1973) ranging from 1 = “Feeling active, vital, alert, or wide awake” to 8 = “Asleep”.

### Statistical Analyses

Statistical analyses generally relied on analyses of variance (ANOVA; SPSS version 21.0.0 for Windows) including a repeated measures factor ‘List length’ (short vs. long) and, the factor ‘Sleep’ (sleep vs. wake). Where appropriate we included additional factors for Time point and Experiment. Significant ANOVA interactions were specified by *post hoc t*-tests. Degrees of freedom were corrected according to Greenhouse-Geisser where appropriate. Whenever using other approaches they are specified next to the results concerned.

## Results

### Memory Task

In Experiment 1 (including the 40 and 160 word pair condition), participants’ absolute retention performance (assessed as the difference in correct words at recall during the Retrieval phase minus at immediate recall during the Learning phase) varied in dependence on list length and sleep/wake group (List length × Sleep interaction: *F*_(1,46)_ = 4.79, *p* = 0.034, η^2^ = 0.094, **Figures 1B,C**). *Post hoc* comparisons revealed that this interaction was mainly driven by better retention performance in the sleep group on the long list of words (*t*_(46)_ = 2.93, *p* = 0.005), an effect that was not evident for the short list (*p* = 0.19). The analysis also yielded main effects indicating better retention performance in the sleep group (*F*_(1,46)_ = 10.84, *p* = 0.002, η^2^ = 0.19) and lower retention performance for the long list (*F*_(1,46)_ = 36.77, *p* ≤ 0.001, η^2^ = 0.44). In Experiment 2, there was no interaction effect of sleep and list length (*F*_(1,51)_ ≤ 1, *p* = 0.78, **Figures 1B,C**) and no main effect of sleep (*F*_(1,51)_ ≤ 1, *p* = 0.62). However, as in Experiment 1, participants retained less associations of the long list (*F*_(1,51)_= 36.49, *p* ≤ 0.001, η^2^ = 0.42). There were no differences between the sleep and sleep deprivation group in Experiment 1 or 2 in the number of correctly recalled word-pairs at immediate recall testing during the Learning phase, used as a measure of encoding (all *p* > 0.59). In both experiments the long list led to the encoding of more word-pairs than the short list (Experiment 1: *F*_(1,46)_ = 239.29, *p* ≤ 0.001, η^2^ = 0.84; Experiment 2: *F*_(1,46)_ = 154.16 *p* ≤ 0.001, η^2^ = 0.75) and the long list in Experiment 2 led to the encoding of more word-pairs than the long list in Experiment 1 (*F*_(1,97)_ = 29.25, *p* ≤ 0.001, η^2^ = 0.23). For the short word-pair lists there was no difference in encoding evident between the experiments (*F*_(1,97)_ = 1.04, *p* = 0.31). **Figure 1D** additionally shows individual data at immediate and delayed recall plotted against each other.

To compare both experiments we did not use a mixed design ANOVA, as in this case our repeated measures ‘List-length’ factor had three levels (40, 160, and 320 word-pairs) that were only incompletely assessed in each participant (i.e., the short list condition was always 40 word-pairs and the long-list condition was either 160 or 320 word-pairs). Instead we conducted a linear mixed effects model analysis, which allows testing incomplete designs (i.e., accounts for the experiments not examining all possible combinations of the list length condition). This analysis confirmed the interaction between Sleep and List length (*p* = 0.048) and the main effects of List length (*p* ≤ 0.001) and Sleep (*p* = 0.048). *P*-values were obtained employing maximum likelihood criteria and a diagonal covariance structure. This covariance structure is the default for repeated measures linear mixed effect models in SPSS 21 and achieved the best model fit compared to other structures. Due to our analyses of the individual experiments we expected the ‘Sleep’ × ‘List length’ interaction, additionally, calculating the model without this interaction reduced model fit. *Post hoc* comparisons revealed that retention performance was significantly reduced from the 160 to 320 word-pair condition in the sleep group (*t*_(47)_ = 2.02, *p* = 0.049), which was not the case in the wake group (*p* = 0.66). There was also no significant difference in retention performance between the sleep and the wake group for the 320 word-pair condition (*p* = 0.68). Nor was there a difference between the two studies in the sleep (*p* = 0.86) or the wake group (*p* = 0.35) for the 40 word-pair condition. Of note, the two experiments also differed with regards to the timing of learning (i.e., the Learning phase began at 19:25 or 16:25 in the Experiments 1 and 2, respectively) and the time between the beginning of learning and immediate recall (i.e., 1:35 h and 3:15 h in the Experiments 1 and 2, respectively), which was not considered in these analyses.

**FIGURE 2:**
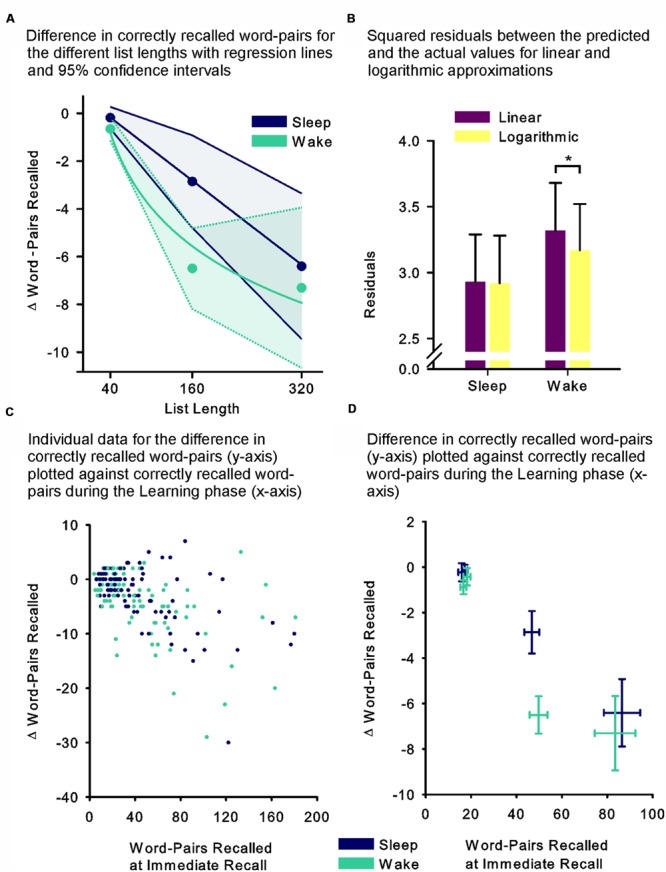
**(A)** Mean differences between recall performance during the Retrieval phase minus performance at immediate recall during the Learning phase, as an estimate of retention performance for the list length conditions of 40 (Experiments 1 and 2), 160 (Experiment 1) and 320 word-pairs (Experiment 2) with linear (sleep – thick blue line) and logarithmic (wake – thick green line) curves fitted (data were assumed to be independent). Thin dashed and full lines, respectively, depict 95% confidence intervals of the means. **(B)** Mean (SEM) residuals between predicted values of the linear (violet fills) and logarithmic (yellow fills) curves and the measured values. **(C)** Individual data for each participant’s performance across the retention interval (difference between recall performance during the Retrieval phase minus performance at immediate recall – y-axis) plotted against his performance during the Learning phase (word-pairs recalled at immediate recall – x-axis) in the sleep (blue) and the wake (green) groups. **(D)** Mean (SEM) retention performance (difference between recall performance during the Retrieval phase minus performance at immediate recall – y-axis) plotted against mean (SEM) performance during the Learning phase (word-pairs recalled at immediate recall – x-axis) for each condition in the sleep (blue) and the wake (green) groups. ^∗^*p* ≤ 0.05.

In an attempt to further specify the dynamics underlying these unexpected results (i.e., the missing effect of sleep for the 320 word-pair condition), we performed an exploratory curve fitting analysis (note that this was not the primary aim of this research). First we visually inspected the means and 95% confidence intervals of the differences in retention performance for the different list lengths, which suggested a linear relationship in the sleep group and a non-linear relationship in the wake group (**Figure 2A**). For statistical analysis we assumed the data to be independent, i.e., we omitted information about repeated measurements as our partially dependent data could not be fitted, which in this case yielded more conservative results. We then fitted linear and logarithmic curves to the sleep and the wake group data separately and compared the regression model containing only the linear predictor (model 1) with a model containing both the linear and the non-linear predictor (model 2). Briefly, we found that model 1 fit the data better in the sleep group, while model 2 demonstrated better fit for the wake group. In detail, for both sleep and wake model 1 containing only the linear predictor explained a significant amount of variance (*F*_(1,96)_ = 31.53, *p* ≤ 0.001, *r*^2^ = 0.247, and *F*_(1,102)_ = 34.71, *p* ≤ 0.001, *r*^2^ = 0.254 for sleep and wake, respectively). Adding the non-linear predictor in the sleep group led to a model 2, which explained the same amount of variance as model 1 (*F*_(2,95)_ = 15.60, *p* ≤ 0.001, *r*^2^ = 0.247), but where none of the predictors individually explained a significant portion of the variance (*p* = 0.22 and *p* = 0.99 for the linear and non-linear predictor, respectively). Adding the non-linear predictor in the wake group led to a model 2, which explained more variance than model 1 (*F*_(2,101)_ = 22.22, *p* ≤ 0.001, *r^2^* = 0.306), importantly, here only the non-linear predictor contributed a significant portion of explained variance (*p* = 0.007), whereas the linear predictor no longer did (*p* = 0.22). To further examine the models we computed the squared residuals between each curve and the measured values of the sleep and the wake groups (**Figure 2B**). The residuals were significantly smaller for the logarithmic than linear curve in the wake group (*t*_(97)_ = 2.40, *p* = 0.018), whereas no difference was found in the sleep group (*p* = 0.91). Correspondingly, an ANOVA including the factors Model (linear vs. logarithmic curve) and Sleep (sleep vs. wake group) revealed a trend towards an interaction (Model x Sleep interaction: *F*_(1,200)_ = 3.76, *p* = 0.054, η^2^ = 0.02). This assessment was similarly supported, when visually inspecting the individual participant’s performance during the Learning phase plotted against retention performance (**Figure 2C**), as well as the mean performance during the Learning phase plotted against the mean retention performance (**Figure 2D**).

### Working Memory Task

Partial load score on the OSPAN task was generally higher during the list Condition performed second (*F*_(1,98)_ = 9.96, *p* = 0.002, η^2^ = 0.09, for main effect of Condition, **Table 1**). There was no main effect of Sleep or Sleep x Condition interaction (all *p* ≥ 0.47). The same structure of results was evident for the OSPAN absolute score (*F*_(1,98)_ = 14.36, *p* ≤ 0.001, η^2^ = 0.13, for main effect of Condition, all other *p* ≥ 0.40). To explore the relationhip between working memory capacity and memory performance, Pearson product-moment correlations were computed separately for each of the list length conditions and sleep or wake groups as well as for the collapsed data. The correlations were calculated for the OSPAN partial load score and absolute score from the respective list length condition, which revealed no significant relationship (*p* ≥ 0.15). Additional analyses were calculated using the OSPAN score from the first Condition, to exclude the sequence effect on OSPAN performance and as working memory is conceptually a trait. Here, independent of sleep and wake there was a trend towards a positive relationship between the OSPAN partial load score and retention of the long list of word-pairs (*r* = 0.19, *p* = 0.065) that was not evident for the absolute score (*r* = 0.06, *p* = 0.50) and did not survive multiple comparison correction. This analysis did not reveal such a relationship independently for the sleep or wake groups in the two experiments for the OSPAN partial load score (*p* ≥ 0.21) or absolute score (*p* ≥ 0.14). Fenn and Hambrick (2012) reported a correlation of *r* = 0.23 (*n* = 255, *p* = 0.02) between working memory capacity and retention performance. Possibly, the effect is not large enough to be found reliably at the current sample size (*n* = 101). Furthermore, whereas Fenn and Hambrick (2012) measured working memory capacity on the evening or morning after the day wake or night sleep retention period, we measured working memory capacity at the same time of day before any sleep manipulation and, therefore, can exclude circadian effects or non-specific effects of the sleep manipulation. Importantly, we found a list length that is highly sensitive to the beneficial effect of sleep on memory, which should also have most robustly reflected any relationship between consolidation and working memory capacity. Hence, limitations in capacity of sleep-dependent memory consolidation seem not to be closely linked to those of working memory capacity.

**Table 1:** Operation span (OSPAN) scores.

	Sleep		Sleep deprivation	
	First	Second	First	Second
**Partial load score**				
Experiment 1	62.41 (1.91)	63.50 (2.01)	59.69 (1.71)	61.08 (2.21)
Experiment 2	57.74 (2.77)	62.26 (2.53)	56.76 (3.29)	61.12 (2.68)
**Absolute score**			
Experiment 1	44.45 (3.07)	48.50 (3.40)	40.96 (2.67)	44.54 (3.78)
Experiment 2	41.33 (3.45)	48.19 (3.43)	39.28 (3.47)	47.08 (3.66)

*Means and SEMs of OSPAN partial load and absolute score.*

### Actimetry

**FIGURE 3:**
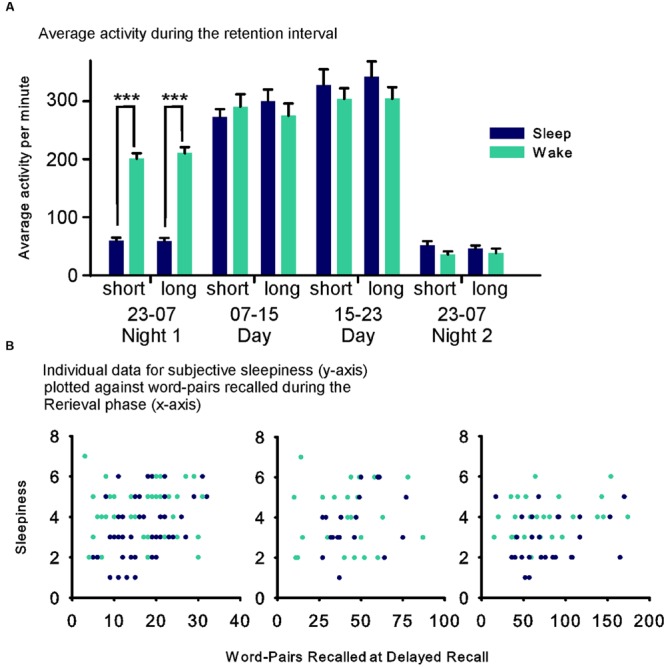
**(A)** Mean (SEM) of the average activity during the four 8 h intervals of the retention interval for the Sleep (blue fills) and Wake (green fills) groups during the short and long list condition, respectively. (Data were comparable and therefore collapsed across Experiments 1 and 2). **(B)** Individual data for each participant’s subjective sleepiness (Stanford Sleepiness Scale – y-axis) plotted against performance during the Retrieval phase (word pairs recalled at delayed recall) for each condition in the sleep (blue) and the wake (green) groups. ^∗∗∗^*p* ≤ 0.001.

The actimetry data were analyzed in four 8 h intervals (**Figure 3**). Data from 8 (2 sleep, 6 wake) participants of Experiment 1 and 13 (7 sleep, 6 wake) participants of Experiment 2 were lost due to recorder malfunction. The analysis across both experiments revealed a Sleep × Time point interaction (*F*_(3,76)_ = 21.816, *p* ≤ 0.001, η^2^ = 0.22) that was mainly driven by higher activity in the wake group between 23:00 h and 07:00 h on the first night after the Learning phase (*F*_(1,76)_ = 273.55, *p* ≤ 0.001, η^2^ = 0.78; **Figure 3**).

### Control Measures

In the word fluency task we found a main effect of Experiment (*F*_(1,95)_ = 6.62, *p =* 0.012, η^2^ = 0.07, **Table 2**) and an interaction of Experiment and List length (*F*_(1,95)_ = 6.62, *p* = 0.012, η^2^ = 0.07). This effect was mainly driven by participants producing more words in the long list condition of Experiment 2 compared to the long list condition of Experiment 1 (*t*_(97)_ = -3.56, *p* ≤ 0.001). This may be due to the high amount of words (640) learned in in the long list condition of Experiment 2 priming an increased reservoir of words that could be produced on this task, or to a general activation of word processing areas of the brain. However, this effect does not explain the absence of a sleep effect in the 320 word-pair condition, as it was similarly observed in the sleep and wake group.

**Table 2:** Control measures.

	Sleep		Sleep deprivation	
	Short list	Long list	Short list	Long list
**Word fluency test**			
Experiment 1 (40/160)	18.05 (0.72)	16.92 (0.55)	17.39 (0.70)	16.62 (0.77)
Experiment 2 (40/320)	18.20 (0.58)	18.83 (0.77)	18.35 (0.73)	19.65 (0.61)
**Reaction speed**		
Experiment 1 (40/160)			
After learning	3.20 (0.09)	3.34 (0.07)	3.19 (0.06)	3.22 (0.07)
After immediate recall	3.16 (0.09)	3.25 (0.08)	3.06 (0.06)	3.14 (0.09)
After retrieval	3.21 (0.07)	3.23 (0.09)	3.05 (0.08)	3.10 (0.08)
Experiment 2 (40/320)			
After learning	3.21 (0.06)	3.31 (0.05)	3.23 (0.06)	3.17 (0.07)
After immediate recall	3.16 (0.08)	3.25 (0.06)	3.20 (0.06)	3.18 (0.08)
After retrieval	3.27 (0.06)	3.27 (0.04)	3.19 (0.06)	3.22 (0.07)
**SSS**		
Experiment 1 (40/160)			
After learning	3.41 (0.33)	3.41 (0.23)	3.15 (0.22)	3.12 (0.26)
After immediate recall	4.18 (0.27)	4.05 (0.27)	4.12 (0.23)	3.85 (0.24)
After retrieval	3.50 (0.31)	3.36 (0.31)	4.77 (0.23)	4.08 (0.33)
Experiment 2 (40/320)				
After learning	3.00 (0.20)	2.74 (0.20)	2.81 (0.20)	3.00 (0.15)
After immediate recall	4.33 (0.24)	4.00 (0.19)	3.77 (0.19)	3.96 (0.19)
After retrieval	3.44 (0.27)	2.96 (0.24)	4.04 (0.23)	3.96 (0.20)

*Means and SEMs for word fluency task, psychomotor vigilance task (reaction speed) and Stanford sleepiness scale (SSS).*

On the psychomotor vigilance tasks participants generally performed faster in the long than short list condition (*F*_(1,93)_ = 4.19, *p* = 0.044, η^2^ = 0.04) and slower after the recall tests both during the Learning phase and during the Retrieval phase (*F*_(2,186)_ = 7.07, *p* = 0.002, η^2^ = 0.07). Also, participants were faster after recall testing in Experiment 2 than 1 (Time point × Experiment: *F*_(2,186)_ = 4.19, *p* = 0.021, η^2^ = 0.04).

Likewise, sleepiness was lower in the long than short list condition (*F*_(1,97)_ = 4.38, *p* = 0.039, η^2^ = 0.04, **Table 1**) and higher after both recall tests (in the Learning phase and Retrieval phase) than after learning in the Learning phase (*F*_(2,194)_ = 44.83, *p* ≤ 0.001, η^2^ = 0.32). Sleepiness was also rated as higher in the wake group on the morning of retrieval testing (*F*_(1,194)_ = 17.59, *p* ≤ 0.001, η^2^ = 0.15).

Assessments of vigilance and sleepiness did not provide any hints that the observed limitations of the sleep induced improvement in retrieval with increased list length might origin from a non-specific effect of the sleep manipulation. The only sleep-related effect was on rated sleepiness, which was increased in the wake group in the Retrieval phase. However, this was not the case for the objective vigilance measure, and rated sleepiness was not significantly correlated with recall performance in the Retrieval phase (**Figure 3B**). Notably, sleepiness was increased generally for the wake groups, which makes it unlikely that this affected the differential effects reported for the word-list lengths conditions. Reduced sleepiness and increased reaction speed in the long compared with the short list condition, could reflect that performing the reaction time task instead of learning word-pairs in the short list condition was more tiring for the participants. However, this difference in reaction speed was already observed in the Learning phase where there were no differences in immediate recall performance between groups and experiments and, hence, cannot explain the differential effects for the longer list lengths selectively at recall during the Retrieval phase.

## Discussion

This study investigated whether there is a limit to the beneficial effect of sleep on declarative memory consolidation. Compared with post-learning performance of the wake control groups, sleep substantially enhanced retention for the 160 word-pair condition, whereas in the 40 word-pair and 320 word-pair conditions no significant effect of sleep was found. This result speaks for the notion that consolidation during sleep is mediated by processes of limited capacity. However, there was no evidence that the effect of sleep on memory retention was in any way linked to working memory capacity.

Immediate recall during the Learning phase reproduced the classical finding that there is an increased number of paired-associates correctly recalled with increasing list length numbers, with this relationship typically following a non-linear function (Tulving and Pearlstone, 1966). The relatively reduced amount of paired associates reproducibly encoded with long lists, is commonly attributed to pro-active interference (Keppel et al., 1968). Here we tried to diminish such interference by including breaks of approximately 16 min after each block of 40 word-pairs. This seems effective, as immediate recall performance for the 320 word-pairs list was indeed on average almost twice as high as for the 160 word-pairs. Nevertheless, residual interference at encoding has likely also occurred in the present experiments.

Forgetting is the opposite of retention, defined here by the difference in recall during the Retrieval phase minus immediate recall performance at Learning. Consistent with previous studies we found that forgetting increases with increased list length (Robinson and Heron, 1922; Robinson and Darrow, 1924). Similar to these reports in our experiments, this relationship appeared to follow a non-linear function, as the amount of forgotten word-pairs seemed to be relatively diminished for the 320 word-pair conditions as compared to the 160 word-pair condition, in the wake condition. This means, although participants at immediate recall (as an estimate of the encoded information) remembered almost twice as many word-pairs in the 320 than in the 160 word-pair condition (on average 83.46 vs. 49.73 word-pairs), the amount of word-pairs forgotten up till the Retrieval phase was only slightly higher (–7.31 vs. –6.50 word-pairs). This pattern argues against a passive decay of traces being the only source of forgetting, as this would result in forgetting proportional to the amount of encoding.

The central result of this study is that the forgetting curve differed depending on whether participants slept or remained awake on the first post-learning night, i.e., sleep substantially enhanced retention specifically in the 160 word-pair condition, but not in the short 40 word-pair and longer 320 word-pair conditions. That sleep did not prove effective in the 40 word-pair condition might surprise, but very likely reflects a ceiling effect, because forgetting in this condition was also marginal in the wake groups (on average < 0.8 word-pairs). The missing benefit from sleep in the 320 word-pair condition is of note. If sleep solely protected the learned word-pairs from retroactive interference, the benefit from sleep for the 320 word-pair condition is expected to be even greater than for the 160 word-pair condition (i.e., the unchanged relative amount of word-pairs affected by interference in the wake group would lead to a greater absolute detriment compared to the sleep group in the 320 word-pair than in the 160 word-pair condition). However, rather than augmenting the effect of sleep, the increase in list length even reduced the effect. This outcome indicates that the brain’s capacity to enhance hippocampus-dependent memory during sleep is basically limited.

At the neurophysiological level the effect of sleep on memory is achieved by the coordinated activity of the three hallmark electrical brain oscillations of SWS (Staresina et al., 2015). The neocortical slow oscillation (∼0.75 Hz) exerts a top-down control on thalamocortical sleep spindles (12-15 Hz) and hippocampal sharp wave-ripples, enabling the replay and feedback of freshly encoded memories, thereby strengthening and transforming them (Feld and Born, 2012). Importantly, enhancing any of these oscillations benefits the consolidation of hippocampus-dependent memory tasks (e.g., Ngo et al., 2013; McNamara et al., 2014). However, recent evidence suggests that this modulation has physiological limits, i.e., sleep spindles show refractoriness (Ngo et al., 2015) and auditory cueing enhances the replay of the cued representation, while overall replay activity remains unchanged (Bendor and Wilson, 2012). While the theory of active systems consolidation during sleep (Diekelmann and Born, 2010) predicts that retention performance should not increase further after a certain level is reached and neurophysiological limitations may indeed prove to be at the heart of this effect, it does not predict that the enhancing effect of sleep on memory should vanish for high loads of information.

The comparison of sleep effects between the 160 and 320 word-pair condition does not only indicate a limit of sleep’s capacities to enhance memory (i.e., no further increase in the sleep induced memory benefit from the 160 to the 320 word-pair condition) but even points towards a reduced memory effect of sleep for the longer list length condition, which is not compatible with the theory of active systems consolidation without considering additional processes of active forgetting. Our additional curve fitting analyses highlight this, as they suggest a linear relationship between list length and forgetting for the sleep group, whereas a logarithmic relationship was evident in the wake group. Older studies only examining wake retention intervals likewise point towards a logarithmic rather than a linear relationship (Robinson and Heron, 1922; Robinson and Darrow, 1924). Extrapolating from our curve analysis, at even larger list length the wake group might show less forgetting than the sleep group. At the neurophysiological level, the linear forgetting curve observed in the sleep group is well in line with the predictions of a sleep-dependent process of global non-specific forgetting (established, for example, via a global synaptic renormalization) that is not limited in capacity and runs independently of the local limited-capacity processes of sleep-dependent strengthening of neuronal memory traces (Born and Feld, 2012; Tononi and Cirelli, 2014). Alternatively, trace-specific down-scaling processes might even be at the core of the reported effect, if one assumes that the memory enhancing effect of sleep is a result of down-selection processes (Tononi and Cirelli, 2014) that have nothing to select at high information loads.

In fact, active processes of forgetting might be established during sleep (Hardt et al., 2013) partly depending on the extent of interference, i.e., overlap in the neural representations of the learned materials (Lewis and Durrant, 2011). Indeed, it could be argued that the 320 word-pair condition introduced intra-list interference that countered the enhancing effects of sleep on memory traces. Yet, as mentioned, signs of interference appeared to be rather diminished than enhanced in our study. Moreover, several previous studies showed that the enhancing effect of sleep on memories goes along with a recovery of memories also from the weakening effect of retroactive interference (Fenn et al., 2003; Drosopoulos et al., 2007), albeit using short lists. A greater rather than smaller effect of sleep on memory with increased list length is also expected if one assumes that learning more word-pairs automatically leads to weaker individual associations, as previous studies showed more robust benefits from sleep for weakly than strongly encoded items (e.g., Schmidt et al., 2006; Drosopoulos et al., 2007).

Unfortunately some actimetry data were lost due to recorder malfunction. Although, the wake group was prevented from sleeping in the first night by staying at the lab, we cannot exclude that some participants slept during the ensuing day. However, the same number of data sets was lost in the wake group for both experiments and the existing data do not suggest that this was a general problem.

## Conclusion

The present findings of a distinct sleep-dependent benefit in memory for a list of 160 word-pairs but not of 320 word-pairs indicate a limitation of capacity for memory consolidation during sleep. This limit is consistent with theories of active systems consolidation during sleep, but speaks against a mere passive role of sleep for protecting memory from interference. Furthermore, clues from our data that for the 320 word-pair condition the sleep-dependent benefit in memory is even significantly lower than for the 160 word-pair condition, i.e., that the sleep and the wake group no longer differ, suggest that with amounts of information close to the capacity limit of the consolidation process, additional processes may be activated that favor forgetting of the learnt materials. This recruitment of active forgetting may reflect that under increased informational load the effect of sleep switches from merely strengthening encoded memory traces to essentially abstracting the memories’ gist by enhancing general features and deleting specific details (Lewis and Durrant, 2011; Westermann et al., 2015).

## Author Contributions

GBF and JB developed the study concept and designed the experiment. PPW ran the experiment and conducted preliminary analyses under supervision of GBF. GBF and JB conducted the final analyses and wrote the manuscript, the methods and results section was based on a first draft by PPW. All authors approved the final version of the manuscript for submission.

## Conflict of Interest Statement

The authors declare that the research was conducted in the absence of any commercial or financial relationships that could be construed as a potential conflict of interest.
